# Initial blood pH during cardiopulmonary resuscitation in out-of-hospital cardiac arrest patients: a multicenter observational registry-based study

**DOI:** 10.1186/s13054-017-1893-9

**Published:** 2017-12-21

**Authors:** Jonghwan Shin, Yong Su Lim, Kyuseok Kim, Hui Jai Lee, Se Jong Lee, Euigi Jung, Kyoung Min You, Hyuk Jun Yang, Jin Joo Kim, Joonghee Kim, You Hwan Jo, Jae Hyuk Lee, Seong Youn Hwang

**Affiliations:** 1 Department of Emergency Medicine, Seoul Metropolitan Government - Seoul National University Boramae Medical Center, 20, Boramae-ro 5-gil, Dongjak-gu, Seoul 07061 Republic of Korea; 2 Department of Emergency Medicine, Gachon University Gil Medical Center, 21, Namdong-daero 774 Beon-gil, Namdong-gu, Incheon, 21565 Republic of Korea; 3 Department of Emergency Medicine, Seoul National University Bundang Hospital, 82, Gumi-ro 173 Beon-gil, Bundang-gu, Seongnam-si, Gyeonggi-do 13620 Republic of Korea; 4 Department of Emergency Medicine, Samsung Changwon Hospital, Sungkyunkwan University School of Medicine, 158, Palyong-ro, Masanhoewon-gu, Changwon-si, Gyeongsangnam-do 51353 Republic of Korea

**Keywords:** Out-of-hospital cardiac arrest, Cardiopulmonary resuscitation, pH

## Abstract

**Background:**

When an out-of-hospital cardiac arrest (OHCA) patient receives cardiopulmonary resuscitation (CPR) in the emergency department (ED), blood laboratory test results can be obtained by using point-of-care testing during CPR. In the present study, the relationship between blood laboratory test results during CPR and outcomes of OHCA patients was investigated.

**Methods:**

This study was a multicenter retrospective analysis of prospective registered data that included 2716 OHCA patients. Data from the EDs of three university hospitals in different areas were collected from January 2009 to December 2014. Univariate and multivariable analyses were conducted to elucidate the factors associated with survival to discharge and neurological outcomes. A final analysis was conducted by including patients who had no prehospital return of spontaneous circulation and those who underwent rapid blood laboratory examination during CPR.

**Results:**

Overall, 2229 OHCA patients were included in the final analysis. Among them, the rate of survival to discharge and a good Cerebral Performance Categories Scale score were 14% and 4.4%, respectively. The pH level was independently related to survival to hospital discharge (adjusted OR 6.287, 95% CI 2.601–15.197; *p* < 0.001) and good neurological recovery (adjusted OR 15.395, 95% CI 3.439–68.911; *p* < 0.001). None of the neurologically intact patients had low pH levels (< 6.8) or excessive potassium levels (> 8.5 mEq/L) during CPR.

**Conclusions:**

Among the blood laboratory test results during CPR of OHCA patients, pH and potassium levels were observed as independent factors associated with survival to hospital discharge, and pH level was considered as an independent factor related to neurological recovery.

## Background

Out-of-hospital cardiac arrest (OHCA) patients whose arrests were witnessed by a bystander, who underwent cardiopulmonary resuscitation (CPR) by a layperson, and who experienced early defibrillation owing to initial shockable rhythm are expected to survive after resuscitation [1, 2]. Consequently, in the case of OHCA patients with the appropriate post-cardiac arrest care, such as goal-directed therapy, early coronary intervention for cardiac protection, and therapeutic hypothermia for brain protection, good outcomes are anticipated. Nevertheless, under some circumstances, it is difficult to make the decision to sustain or stop CPR in OHCA patients, which raises difficult problems for health care providers. Per the recent advanced cardiovascular life support (ACLS) guidelines, the duration of effort put forth for resuscitation should be based on an individual’s situation, which also depends on the CPR leader’s judgment. In addition, the condition and outcome of the OHCA patient during CPR are often not clear. Frequently, unnecessary CPR has been performed without having definitive information about the status of the OHCA patient.

When OHCA patients are administered ACLS, the peripheral venous line should be secured because of the administration of epinephrine. The initial status of patients can be estimated using a blood sample obtained during CPR. Moreover, the results of using point-of-care testing (POCT), such as arterial or venous blood gas analysis, can be quickly observed. However, the exact means for obtaining the result of blood sampling during CPR is unknown. There are some reported studies on the prediction of the outcome of OHCA patients during CPR using blood laboratory tests or Utstein-style variables [3–5]. There are some limitations with respect to the applicability of these studies to OHCA patients owing to small sample size, use of only a single center, or lack of validation. In the present study, the relationship between the initial results of blood laboratory tests during CPR and outcomes of OHCA patients was investigated using multicenter, large-cohort data.

## Methods

### Study design, setting, and population

We conducted a retrospective analysis of prospective registered data of OHCA patients who were admitted to the emergency department (ED) in three hospitals between January 2009 and December 2014. Three hospitals participated in this study, and approval for this study was obtained from the institutional review board of each hospital. All of the hospitals are tertiary university hospitals and located in different large urban areas, specifically Seoul (site A), Incheon (site B), and Seongnam (site C). The ED volume of each hospital is approximately 60,000, 100,000, and 80,000 patients per year, respectively. All of the hospitals were equipped with facilities, equipment, and medical personnel to provide the final intensive treatment to OHCA patients. Each hospital obtained approval from their respective institutional review board for data collection and follow-up of OHCA patients under the waiver of informed consent granted by the ethics committee. All of the hospitals have a prospective OHCA registry according to the standardized Utstein-style guideline. The information for all OHCA patients was collected after CPR was administered and recorded in each hospital’s OHCA registry. We included nontraumatic OHCA patients over the age of 18 years. Patients who already had a prehospital return of spontaneous circulation (ROSC) upon arrival at the ED as well as traumatic OHCA patients were excluded. Patients for whom blood gas analysis during CPR could not be obtained were also excluded from the analysis. Variables included in the prospective registry were as follows: age, sex, call-to-hospital arrival time, ACLS time in the ED, witnessed arrest, presumed origin of arrest, bystander CPR, initial rhythm in the ED, targeted temperature management (TTM), emergent coronary angiography (E-CAG), emergent percutaneous coronary intervention (E-PCI), sustained ROSC, survival to hospital discharge, and Cerebral Performance Categories Scale (CPC) score at 1 month. Good and poor neurological outcomes were defined as CPC 1–2 and CPC 3–5, respectively. The retrospectively identified variables of the initial blood laboratory test results during CPR were as follows: sodium, potassium, chloride, total CO_2_ level, glucose, lactate, pH, partial pressure of carbon dioxide (pCO_2_), partial pressure of oxygen (pO_2_), and bicarbonate (HCO_3_^−^). All blood samples were drawn during the early CPR phase (within 5 minutes) after arrival at the ED. In addition, all blood samples were sent to the central laboratory and analyzed. Only the most rapidly analyzed blood samples were included in the final data collection. We excluded the results of the blood samples that were drawn during the late CPR phase (over 5 minutes), after ROSC, or during repeated resuscitation. We also excluded pCO_2_, pO_2_, and HCO_3_^−^ from the results of the blood gas analysis in the final multivariable logistic analysis because of disagreement between the arterial and venous blood. Blood gas analyses were performed using the RAPIDPoint 405 (Siemens Medical Solutions, Munich, Germany) at site A, ABL90 (Radiometer Medical ApS, Copenhagen, Denmark) at site B, and GEM Premier 3000 (Instrumentation Laboratory, Bedford, MA, USA) at site C.

### Statistical analysis

Continuous data were expressed as the medians and IQRs according to a normal distribution. Categorical data were expressed as numbers and percentages. The Mann-Whitney *U* test and chi-square test were used for univariate comparisons of the baseline characteristics between the CPR outcomes [6]. The statistically significant variables from the univariate analysis (*p* < 0.05) and clinically important variables were included in the final multivariable logistic regression model that was conducted in a backward stepwise manner. Missing data that exceeded 10% for any variable were not considered in the multivariable logistic analysis. As such, the lactate level was excluded from the final analysis because the missing data exceeded 10%. A multivariable logistic regression model analysis was performed to estimate the OR of survival to hospital discharge and good neurological recovery at 1 month with 95% CI. The calibration was assessed by comparing the expected and observed survival to hospital discharge rates and good neurological recovery using the Hosmer-Lemeshow goodness-of-fit test. A *p* value < 0.05 was considered to represent an inadequate model fit. Among the statistically significant blood laboratory test variables in the multivariable logistic analyses for survival to hospital discharge and good neurological recovery, additional analyses were performed after categorizing the pH levels (scored from 1 to 8; ≤ 6.7, 6.701–6.800, 6.801–6.900, 6.901–7.000, 7.001–7.100, 7.101–7.200, 7.201–7.300, ≥ 7.301) and serum potassium levels (scored from 1 to 7; ≥ 10.01, 8.51–10.00, 7.01–8.50, 5.51–7.00, 3.51–5.50, 2.51–3.50, ≤ 2.5) according to the rate of good neurological recovery because of the presence of a nonlinear trend exhibited by the potassium levels. The pH + K^+^ score was calculated by adding the pH and potassium scores, which ranged from 1 to 15 points. The multivariate logistic analysis included the laboratory variables of the pH and potassium levels. A *p* value < 0.05 was assumed to indicate statistical significance. The data were analyzed using IBM SPSS Statistics version 20.0 software (IBM, Armonk, NY, USA). The predictive ability of the variables was examined by calculating the ROC curves with their responding AUCs.

## Results

### Enrolled patients and outcomes

**Fig. 1:**
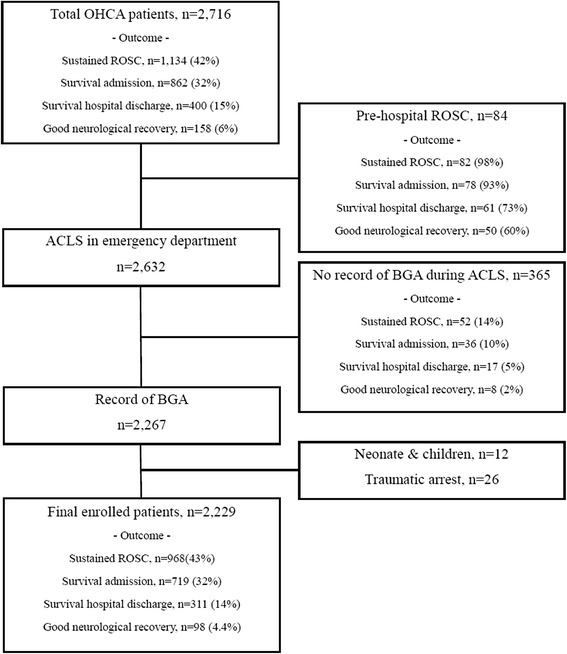
Study population and outcomes of out-of-hospital cardiac arrest (OHCA). *ACLS* Advanced cardiac life support, *ROSC* Return of spontaneous circulation, *BGA* Blood gas analysis

A total of 2716 OHCA patients who visited the EDs of three hospitals during the study period were reviewed. In total, 996 OHCA patients visited site A, 770 visited site B, and 950 visited site C. All of the patients except those who had a prehospital ROSC underwent CPR in the ED. The proportion of the outcomes among the 2716 OHCA patients is provided in Fig. 1. Four hundred ninety patients were excluded from the present study, and 2229 OHCA patients were enrolled in the study. The final numbers of enrolled OHCA patients at each hospital were 770, 661, and 798 at sites A, B, and C, respectively. The percentages of patients who recovered to sustained ROSC, were admitted to the hospital, survived to discharge, and had good neurological recovery (CPC 1–2) were 43% (968 patients), 32% (719 patients), 14% (311 patients), and 4.4% (98 patients), respectively.

A comparison of blood laboratory test results according to survival to discharge or good neurological recovery is shown in Table 1.

**Table 1:** Baseline characteristics for survival to hospital discharge and good neurological recovery

	Survival to hospital discharge			Good neurological recovery		
	Yes (*n* = 311)	No (*n* = 1918)	*p* Value	Yes (*n* = 98)	No (*n* = 2131)	*p* Value
Sex			0.015			0.01
Male, *n* (%)	219 (15.3)	1214 (84.7)		75 (5.2)	1358 (94.8)	
Female, *n* (%)	92 (11.6)	704 (88.4)		23 (2.9)	773 (97.1)	
Age, years, mean (IQR)	59 (49–73)	66 (51–76)	0.001	55 (46–65)	66 (51–76)	< 0.001
Prehospital CPR duration, minutes, mean (IQR)	20 (14–25)	23 (13–30)	< 0.001	16 (9–24)	23 (12–30)	< 0.001
ACLS duration, minutes, mean (IQR)	12 (6–20)	21 (13–30)	< 0.001	10 (4–21)	20 (12–30)	< 0.001
Witnessed arrest, *n* (%)	214 (68.8)	1054 (55)	< 0.001	82 (83.7)	1186 (55.7)	< 0.001
Cardiac origin, *n* (%)	123 (39.5)	719 (38.9)	0.486	58 (59.2)	784 (36.8)	< 0.001
Bystander CPR, *n* (%)	129 (41.5)	747 (38.9)	0.396	49 (50)	827 (38.8)	0.027
Shockable rhythm (ED), *n* (%)	68 (21.9)	149 (7.8)	< 0.001	51 (52)	166 (7.8)	< 0.001
TTM, *n* (%)	160 (51.4)	208 (10.8)	< 0.001	57 (58.2)	311 (14.6)	< 0.001
Emergent CAG, *n* (%)	46 (14.8)	55 (2.9)	< 0.001	29 (29.6)	72 (3.4)	< 0.001
Emergent PCI, *n* (%)	29 (9.3)	32 (1.7)	< 0.001	20 (20.4)	41 (1.9)	<0.001
pH, mean (IQR)	7.00 (6.93–7.31)	6.96 (6.83–7.20)	< 0.001	7.11 (7.00–7.26)	6.96 (6.84–7.09)	< 0.001
pCO_2_ (mmHg), mean (IQR)	70 (47–88)	72 (54–93)	0.01	60 (39–74)	72 (54–93)	< 0.001
pO_2_ (mmHg), mean (IQR)	48 (17–87)	30 (15–59)	< 0.001	53 (21–84)	30 (15–61)	< 0.001
HCO_3_^−^ (mEq/L), mean (IQR)	19 (15–22)	17 (13–21)	< 0.001	17 (14–22)	17 (13–21)	0.308
Lactate (mmol/L), mean (IQR)	9.5 (6.9–11.7)	10.1 (7.1–13.6)	0.006	8.7 (6.8–10.8)	10.1 (7.1–13.4)	0.011
Sodium (mmol/L), mean (IQR)	140 (136–142)	140 (136–143)	0.685	140 (136–142)	140 (136–143)	0.974
Potassium (mmol/L), mean (IQR)	4.9 (4.0–6.0)	6.9 (4.7–7.4)	< 0.001	4.3 (3.7–5.3)	5.8 (4.6–7.2)	< 0.001
Chloride (mmol/L), mean (IQR)	105 (100–108)	104 (99–108)	< 0.071	106 (101–109)	104 (99–108)	0.016
Total CO_2_ (mmol/L), mean (IQR)	18 (14–22)	16 (11–20)	< 0.001	17 (14–20)	16 (11–20)	0.014
Glucose (mg/dL), mean (IQR)	223 (152–304)	215 (122–309)	0.044	197 (152–268)	217 (126–310)	0.707

*Abbreviations: CPR* Cardiopulmonary resuscitation, *ACLS* Advanced cardiovascular life support, *ED* Emergency department, *TTM* Targeted temperature management, *CAG* Coronary angiography, *PCI* Percutaneous coronary intervention, *HCO*_*3*_^*−*^ Bicarbonate, *pCO*_*2*_ Partial pressure of carbon dioxide, *pO*_*2*_ Partial pressure of oxygen

**Fig. 2:**
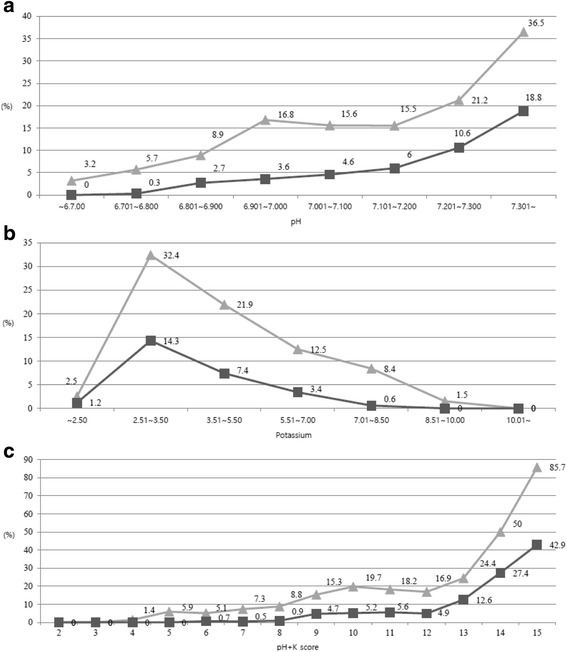
Outcomes according to the initial pH level, potassium level, and pH + K^+^ score during advanced cardiovascular life support. **a** pH. **b** Potassium. **c** pH + K^+^ score. *Gray line* = survival to hospital discharge, *black line* = good neurological recovery

There were statistically significant differences in all of the Utstein-style variables and variables related to post-cardiac arrest care (E-PCI, TTM) for good neurological recovery. In addition, statistically significant differences in the pH, pCO_2_, pO_2_, lactate, potassium, and total CO_2_ between the good and bad outcomes were observed. The relationship between outcome and each blood laboratory test result is shown in Fig. 2.

The pH levels derived from the blood gas analysis exhibited a statistically significant relationship with survival to hospital discharge and good neurological recovery. Typically, patients with good neurological recovery had a pH level ≥ 6.8. As potassium levels increased beyond the normal range, the proportion of patients with good neurological recovery decreased. No patients who survived to hospital discharge had potassium levels > 10 mEq/L. None of the patients with good neurological recovery had potassium levels > 8.5 mEq/L. Results of multivariable logistic regression analysis for survival to hospital discharge and good neurological recovery are shown in Table 2.

**Table 2:** Multivariable logistic analysis for survival to hospital discharge and good neurological recovery

	Survival to hospital discharge		Good neurological recovery	
	OR (95% CI)	*p* Value	OR (95% CI)	*p* Value
Male sex	1.536 (1.121–2.104)	0.008		
Age, years	0.990 (0.982–0.999)	0.032	0.961 (0.946–0.977)	< 0.001
Call-to-hospital arrival time	0.979 (0.965–0.993)	0.003	0.950 (0.921–0.980)	0.001
ACLS time	0.952 (0.938–0.966)	< 0.001	0.956 (0.929–0.982)	0.001
Witnessed arrest			2.814 (1.406–5.632)	0.003
Shockable rhythm in ED	1.829 (1.194–2.802)	0.006	8.111 (4.632–14.203)	< 0.001
TTM	5.860 (4.332–7.928)	< 0.0001	7.546 (4.406–12.924)	< 0.001
E-PCI			4.442 (1.940–10.170)	< 0.001
E-CAG	2.316 (1.345–3.988)	0.002		
pH	6.287 (2.601–15.197)	< 0.001	15.395 (3.439–68.911)	< 0.001
Potassium	0.888 (0.821–0.962)	0.003		

*Abbreviations: ACLS* Advanced cardiovascular life support, *ED* Emergency department, *TTM* Targeted temperature management, *E-CAG* Emergent coronary angiography, *E-PCI* Emergent percutaneous coronary intervention

**Fig. 3:**
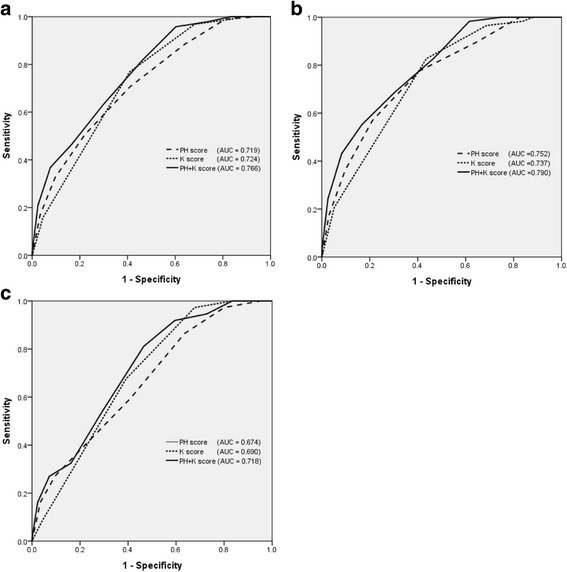
ROC curves of pH score, potassium score, and pH + K^+^ score for good neurological recovery. **a** Total patients. **b** Patients with presumed cardiac origin. **c** Patients with presumed noncardiac origin

Sex, age, call-to-hospital arrival time, ACLS time, initial rhythm in ED, TTM, E-CAG, potassium, and pH were observed as statistically significant variables for survival to hospital discharge. Age, call-to-hospital arrival time, ACLS duration, witnessed arrest, initial rhythm in ED, TTM, E-PCI, and pH were observed as statistically significant variables for good neurological recovery. pH was observed as a strong variable for good neurological recovery in all multivariable analytical models (OR 15.395, 95% CI 3.439–68.911; *p* < 0.001). ROC curves of the blood laboratory test results for the prediction of good neurological recovery are shown in Fig. 3.

**Fig. 4:**
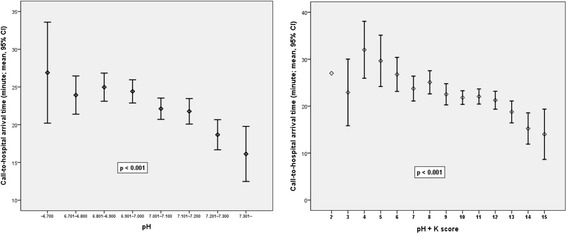
The change of call-to-hospital arrival time according to the pH score and pH + K^+^ score in witnessed out-of-hospital cardiac arrest patients

The ROC analysis revealed that the AUCs of the pH and potassium scores for good neurological recovery were 0.719 and 0.724, respectively. The AUC of the pH + K^+^ score was 0.766 (95% CI 0.722–0.809). When the presumed cardiac origin (842 patients) and presumed noncardiac origin groups (1387 patients) were analyzed separately, the AUCs of the pH, potassium, and pH + K^+^ scores were found to be higher in the cardiac origin group than in the noncardiac origin group (0.752, 0.737, and 0.790 versus 0.674, 0.690, and 0.718, respectively). The association between pH and prehospital CPR duration is shown in Fig. 4.

Only the OHCA patients whose exact call-to-hospital arrival time was known with a witness were included in this analysis (*n* = 1220). The shorter call-to-hospital arrival time reduced the likelihood of survival to hospital discharge and good neurological recovery (adjusted ORs 0.980 and 0.941, 95% CIs 0.964–0.997 and 0.911–0.972, *p* < 0.001 and *p* < 0.001, respectively). There was a statistically significant association between pH and call-to-hospital arrival time (*p* < 0.001). None of the patients who had a good neurological recovery had a call-to-hospital arrival time > 44 minutes. There was also a statistically significant relationship between the pH + K^+^ score and call-to-hospital arrival time (*p* < 0.001).

## Discussion

This study is the first report of an investigation of the association between blood laboratory test results during CPR and OHCA outcomes using large-cohort data. There was a statistically significant association between some blood laboratory test results during CPR and OHCA outcomes. The pH and potassium levels were significantly related to survival to hospital discharge, and the pH level was significantly associated with good neurological recovery. None of the OHCA patients who had good neurological recovery had a pH level < 6.8 or a potassium level > 8.5 mEq/L during CPR.

There were some limitations in getting information and assessing OHCA patients’ condition during CPR. When OHCA patients were not witnessed in the field, it was difficult for a health care provider to determine whether CPR should be continued or transportation to the hospital should continue. Therefore, many health care providers may have subjectively made the decision about CPR without any objective evidence. In addition, clear guidance about CPR guidelines does not exist [7, 8]. The termination of resuscitation (TOR) protocol can be of help to health care providers or emergency medical service (EMS) personnel [9, 10]. Nevertheless, in Korea, EMS personnel are not authorized to assume or pronounce death in the field [11]. All OHCA patients must be transported to the hospital. Therefore, the emergency medical team makes the decision about the continuation of CPR. If an OHCA patient was transferred to the hospital according to the TOR rule, there was no decision left for the emergency physician to make. It is generally accepted that asystole for > 20 minutes in the absence of a reversible cause and with ongoing ACLS constitutes a reasonable ground for ceasing further resuscitation attempts [12].

A POCT device that is capable of a real-time blood test in the ED has been reported in the literature [13–15]. The device has the potential to provide quick identification of a patient’s condition based on the results. In fact, emergency medical teams typically use this POCT during CPR and evaluate the patient’s status using blood laboratory test results. However, important diagnostic information derived from the POCT results, especially acid-base status, in OHCA patients under CPR was not available. Therefore, we examined the relationship between real-time laboratory test results using the POCT during CPR and outcomes after CPR. In the present study, we used a prospective multicenter OHCA registry with a retrospective review of the laboratory test results.

There have been previous studies on the relationship between laboratory test results during CPR and patient outcomes [3, 4, 16, 17]. Most of those studies were performed after ROSC in OHCA patients [18–22]. In these studies, lactate and ammonia levels during or after CPR were significantly different between the good and bad outcomes in OHCA patients. Researchers in one study reported the relationship between the laboratory test results, which included blood gas analysis during CPR, and the outcomes of CPR [17]. The researchers demonstrated the differences in some biochemical analyses (total protein, potassium, inorganic phosphorus, and ammonia), pH, pCO_2_, pO_2_, HCO_3_^−^, and base excess (BE) on arrival between those with good and bad neurological outcomes. The average pH, pO_2_, and BE in the bad outcome group were lower than among those in the good outcome group. However, there was no laboratory factor independently associated with the outcome after multivariable analysis. Researchers in a study involving 32 patients among 826 OHCA patients reported that severe acidemia on arrival was not predictive of neurological outcome [23]. They demonstrated that the arterial pH on arrival was not significantly associated with neurological outcomes. There was no significant difference in the outcome between the severe acidemia (pH < 7.2) group and the remaining group of post-cardiac arrest patients treated with TTM. The study was a retrospective single-center study with a small cohort. Therefore, on the basis of that study, it was difficult for us to understand the exact meaning of the pH level on arrival. Nevertheless, the authors suggested that a prospective multicenter study is needed to evaluate the predictive value of pH at the time of arrival at the ED regarding neurological outcomes. Recently, a prospective observational study on the role of blood gas analysis during CPR in OHCA patients was published [24]. The researchers investigated the predictive strength of arterial blood gas analysis during CPR regarding sustained ROSC in OHCA patients and reported no significant association between pH and sustained ROSC after multivariate analysis. The pCO_2_ level was associated only with sustained ROSC among the other variables of arterial blood gas analysis. In our study, no relationship between initial pH during CPR and sustained ROSC was observed, and the AUC of pH was 0.553 (95% CI 0.529–0.577) for sustained ROSC. Hence, it is proposed that the initial pH level is not a surrogate marker for the prediction of sustained ROSC during CPR in OHCA patients.

The venous pH has sufficient agreement with the arterial pH. The two are clinically interchangeable [25–27]. Therefore, the pH of randomly sampled blood during CPR was included in the multivariable analyses with the assumption that there is no significant difference in the pH values between arterial and venous blood. We did not include pCO_2_ and pO_2_ in the multivariable analysis, because medical teams did not know exactly whether the artery or the vein was employed as the sampling vessel during CPR. In most of the studies on blood gas analysis during conditions that require critical care, arterial line insertion or a central vein cannula was used. However, the central vein cannula or arterial line insertion is a very difficult procedure during the early CPR phase. There were two recent investigations on arterial blood gas analysis in OHCA patients on arrival at the ED [23, 28]. The proportions of these patients in all OHCA patients who received ACLS in the two studies were 4% (32 of 836) and 13% (83 of 619), respectively. These results signify that the examination of arterial blood gas analysis during CPR is a very difficult task and would inevitably result in selection bias. In real prehospital and hospital CPR situations, the peripheral vein is the most appropriate vessel for blood sampling. CPR drugs must be administered rapidly through a peripheral blood vessel as soon as possible. There is an available clinical evaluation method of the OHCA patient’s status through POCT using the peripheral blood during the early CPR phase. If ultrasonography is available to access the sampling vessel during CPR, blood sampling to obtain accurate arterial or venous blood using the large blood vessels becomes possible.

Outcomes in OHCA patients were absolutely influenced by no flow time and CPR duration [29, 30]. The present study demonstrates that there was a significant relationship between pH and call-to-hospital arrival time in witnessed OHCA patients. A decrease in the pH level was noted according to the increase in the call-to-hospital arrival time. This result signifies that the pH level is a substitute for prehospital CPR duration. In cases of unwitnessed OHCA patients, if the POCT result was obtained during the early CPR phase in the prehospital or hospital area, the data may help health care providers to determine whether to continue the resuscitation. None of the patients who had good neurological recovery had a pH level < 6.8 or a potassium level > 8.5 mEq/L. In addition, there was a very low probability (< 1%) of good neurological recovery in patients with low pH + K^+^ scores (≤ 8 points). The present study also demonstrates that there was a significant relationship between the pH + K^+^ score and call-to-hospital arrival time. If a prediction tool is developed using the call-to-hospital arrival time and laboratory results on admission to the ED, including the meaningful Utstein variables, it will be helpful to health care providers in the ED. If an emergency medical technician can use the POCT device during field CPR, significant aid can be obtained to make the best decision regarding whether to continue resuscitation, and the outcomes can be obtained through further studies.

There is some discrepancy in the reliability between the POCT and central laboratory electrolyte results in critical care management [31–34]. The discrepancies between the POCT (employing plasma) and core laboratory analyzers (employing serum) may occur during critical care management. Unlike normal patients, the electrolyte results of CPR patients have a very wide range. Nevertheless, there have not yet been any investigations about the conflicting outcomes regarding the results during CPR. Therefore, in this study, laboratory analyzers for obtaining results on electrolytes were used. In future studies, the reliability of the electrolyte results between the POCT and laboratory analyzer during CPR should be investigated.

The present study has some limitations. First, this study was a retrospective study initiated using a prospective OHCA registry. Consequently, there were many patients with no record of blood gas analysis and with missing laboratory variables. The reason is the lack of blood sampling volume for analysis and difficult blood sampling during CPR. Second, we were unsure if the blood sample was drawn from an arterial or venous line. Therefore, we did not analyze some of the laboratory results, such as pCO_2_, pO_2_, and lactate, in the multivariable logistic regression analysis. Large-scale prospective studies that use only the blood vessels between the artery and vein during CPR are needed to include pCO_2_ and pO_2_ in the analysis. We considered blood sampling using a peripheral vein because of its simplicity and actual possibility in the clinical situation. In addition, the initial lactate level, which is known to be an important predictor on the basis of a previous study, was not included in the multivariable analysis, owing to the large number of missing values [3]. Third, we could not acquire the exact time of blood sampling from the ED arrival; hence, the adjustment of the pH value according to the blood sampling time could not be performed. In future studies, the time from ED arrival to sampling needs to be considered when analyzing time variables. Fourth, it is dangerous to discontinue CPR, because the pH level of the blood gas analysis performed during CPR alone is < 6.8. The cessation of CPR will require consideration of the patient’s condition along with other information as well as the pH level, and there should be additional prospective large-scale studies with pH and other important variables. Fifth, there were no predefined algorithms to stop CPR in three hospitals. Additional prospective studies should have such an algorithm. Sixth, we did not statistically test a linear relationship between pH level and outcome in logit scale. There were actually 12 cases (0.5%) with higher-than-normal pH levels, and their outcomes tended to decrease.

## Conclusions

In a multicenter, large-scale, registry-based study, pH and potassium levels were found to be significantly associated with survival to hospital discharge, and the pH level was significantly associated with good neurological recovery. The OHCA patients who had good neurological recovery had pH levels ≥ 6.8, and their potassium levels were > 8.5 mEq/L during CPR. Among the blood laboratory test results, the initial pH level during CPR is considered as an independent factor for survival to hospital discharge and neurological recovery in OHCA patients.

## Abbreviations

ACLS: Advanced cardiovascular life support
BE: Base excess
BGA: Blood gas analysis
CAG: Coronary angiography
CPC: Cerebral Performance Categories Scale
CPR: Cardiopulmonary resuscitation
E-CAG: Emergent coronary angiography
ED: Emergency department
EMS: Emergency medical service
E-PCI: Emergent percutaneous coronary intervention
HCO_3_^−^: Bicarbonate
OHCA: Out-of-hospital cardiac arrest
PCI: Percutaneous coronary intervention
pCO_2_: Partial pressure of carbon dioxide
pO_2_: Partial pressure of oxygen
POCT: Point-of-care testing
ROSC: Return of spontaneous circulation
TOR: Termination of resuscitation
TTM: Targeted temperature management
